# THD-Module Extractor: An Application for CEN Module Extraction and Interesting Gene Identification for Alzheimer’s Disease

**DOI:** 10.1038/srep38046

**Published:** 2016-11-30

**Authors:** Tulika Kakati, Hirak Kashyap, Dhruba K. Bhattacharyya

**Affiliations:** 1 Department of Computer Science and Engineering, Tezpur University, Napaam, 784028, India; 2 Department of Computer Science, University of California, Irvine, 92697, USA

## Abstract

There exist many tools and methods for construction of co-expression network from gene expression data and for extraction of densely connected gene modules. In this paper, a method is introduced to construct co-expression network and to extract co-expressed modules having high biological significance. The proposed method has been validated on several well known microarray datasets extracted from a diverse set of species, using statistical measures, such as *p* and *q* values. The modules obtained in these studies are found to be biologically significant based on Gene Ontology enrichment analysis, pathway analysis, and KEGG enrichment analysis. Further, the method was applied on an Alzheimer’s disease dataset and some interesting genes are found, which have high semantic similarity among them, but are not significantly correlated in terms of expression similarity. Some of these interesting genes, such as MAPT, CASP2, and PSEN2, are linked with important aspects of Alzheimer’s disease, such as dementia, increase cell death, and deposition of amyloid-beta proteins in Alzheimer’s disease brains. The biological pathways associated with Alzheimer’s disease, such as, Wnt signaling, Apoptosis, p53 signaling, and Notch signaling, incorporate these interesting genes. The proposed method is evaluated in regard to existing literature.

The volume of data obtained by microarray or other new high throughput technologies have been increasing enormously, and as a result, the biological databases are growing enormously. The key challenge is to analyze these large amount of data and extract meaningful information. Therefore, it is an interesting and challenging task to represent these data in an easy way, so that researchers can communicate and interpret data efficiently. A biological network graph allows interactive visual representation and analysis of data. In a biological network, nodes represent bio-entities (genes or proteins) and edges represent associations between the bio-entities. The biological networks can be categorized broadly based on their constituent bio-entities, such as, protein-protein interaction network, gene co-expression network (CEN), and metabolic network. These biological networks encode the relationships, such as, correlation, co-expression, and co-regulation, among the bio-entities.

A gene CEN is defined as an undirected graph, where two genes are connected to each other if their expression levels are similar across conditions. Information related to physical-interaction, genetic interaction, shared protein domains, co-localization, pathway, and predicted functions can be inferred from a gene CEN^1^. A gene CEN primarily shows correlation or association in a pair of genes in terms of standard correlation measures and mutual information. These networks encode the relationships of each entity with its neighbors, which helps in geometrical interpretations for extraction of functionally related genes or network modules.

There are many established methods for construction of CEN based on various approaches and correlation measures. CEN serves in analysis of functionally related groups of genes, termed as network modules. Therefore, along with the construction of CEN, it is important to extract the functionally correlated network modules. Researchers have also introduced several tools and methods to support extraction of network modules from co-expression networks^2^,^3^,^4^,^5^.

Medical applications of analysis of co-expressed modules are many. One of such applications is to find interesting genes related to a disease. Most of the network based approaches extract co-expressed network modules from a CEN. Analysis of such network modules facilitates the study of molecular differences across different stages of a disease^6^. Methods presented in refs 7, 8, 9, 10, 11 suggest the application of CEN module extraction techniques in the analysis of genes related to diseases, such as Alzheimer’s Disease (AD). These studies consider the co-expressed genes in network modules based on expression similarity.

Gene expression data assess only experimental information, but not the functional associations among genes. However, for biological relevance of results, Gene Ontology (GO) derived information must be incorporated, which encode additional information, such as, functional similarity and co-membership of genes or proteins in a biological pathway^12^. Functional similarity between two genes with annotations can be measured using their semantic similarity. Semantic similarity is based on GO database and describes the topological distance between two terms in the hierarchical taxonomy. Semantic similarity between two genes can be measured on the basis of information content (Ic). Ic is defined as shown in Equation (1).


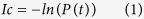


where,


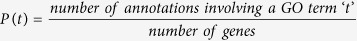


This paper considers the important issue of analysis of CEN using both gene expression similarity and semantic similarity. The proposed method analyzes CEN considering not only the highly co-expressed genes, but also the genes with less expression similarity, yet high semantic similarity, which are termed as border genes in this paper. The border genes obtained are found to be involved in biological pathways, and therefore, are highly functionally related.

The proposed method is applied to find the potential biomarkers related to the progression of AD. Alzheimer’ disease, mostly seen in elderly persons, is related to degeneration of neurons, leading towards dementia. With the growth of data from genomics, proteomics, and interactomics, it has become easy for researchers to understand the dysfunctionalities at molecular level during progression of a disease. In case of AD, pathways, such as *Wnt signaling, Alzheimer’s disease, Apoptosis signaling*, and *Glycolysis*, are related to the progression of the disease. Therefore, understanding of the genes participating in a network module with Pathway analysis, KEGG enrichment analysis, and GO terms helps to identify the biomarkers related to the disease.

Existing methods^9^,^11^,^13^,^14^,^15^, use CEN module extraction techniques that prioritize the genes and study them at molecular level during progression of AD and other neurodegenerative diseases. Alzbase^11^ is an integrated approach, which reveals links between upstream genetic variations and downstream endo-features and prioritizes genes in relation to AD. Method proposed by Yue *et al*.^9^ combines gene pair scores of four existing methods to provide robust and useful results enriched with pathways, such as AD, Parkinson’s disease, and Huntington’s disease.

In this paper, a method named THD-Module Extractor is introduced, which can extract biologically relevant modules from CEN and identify border genes related to AD with less expression similarity and high semantic similarity. The method accepts a microarray gene expression data *M* and two thresholds, namely, expression similarity threshold (*δ*) and minimum neighborhood threshold (*ρ*). The method constructs CEN from gene expression data and extracts highly co-expressed modules.

The proposed method uses SSSim^16^ as the expression similarity measure and Lin^17^ as semantic similarity measure. SSSim finds similarity between genes exhibiting shifting, scaling, and shifting-and-scaling patterns. The SSSim measure is chosen over other correlation measures since SSSim is robust to noisy values. For semantic similarity, the Lin measure is chosen as it is a normalized relative measure that finds the differences in information content of two GO terms being compared^12^.

The effectiveness of the proposed method is validated using various datasets, including AD dataset. In case of AD dataset, the method identifies the border genes involved in pathways related to the progression of the disease. It is observed that the highly differentially expressed border genes share some important pathways, GO terms, and KEGG pathways, known to be related to AD. The border genes are assessed to find their significance in progression of AD. The work flow of THD-Module Extractor, is illustrated in Fig. 1.

This work provides a comprehensive solution towards identifying the biomarkers of diseases, such as AD, based on expression and semantic similarity of genes, obtained from module extraction method. The following are the major contributions of this work.

1. In this paper, a CEN module extraction method named THD-Module Extractor is introduced, which identifies not only the highly co-expressed genes, but also extracts the genes with less expression similarity and high semantic similarity. In contrast, these genes are not considered by any of the existing related methods.
2. The method is validated using datasets pertaining to various species, in terms of both statistical and biological significance measures. For the AD dataset, the genes with less expression similarity and high semantic similarity are assessed using GO, pathway analysis, and KEGG enrichment analysis to prioritize the genes related to the progression of the disease.
3. Correlation analysis of each pair of genes in a large dataset is computationally expensive. This work leverages the parallel computing capabilities of the Graphics Processing Units (GPU) to find the SSSim^16^ correlation matrix, implemented using the NVIDIA CUDA library.

## Results

### Construction of CEN and extraction of network modules

THD-Module Extractor accepts a microarray gene expression dataset and two thresholds, the expression similarity threshold (*δ*) and the minimum neighborhood threshold (*ρ*), to construct CEN and to extract modules with high expression similarity using a similarity measure, SSSim. The method identifies border genes with low expression similarity and high semantic similarity from the highly co-expressed network modules. These border genes are analyzed to identify disease related genes from AD dataset.

The threshold (*δ*) of the module extraction process is gradually updated from 0.9 to 0.5 by a factor *α* in each iteration. The minimum neighborhood threshold (*ρ*) is set to 3 in the conducted experiments. The generated co-expressed modules are validated in terms of *p* and *q* values.

For a list of genes, the *p* value represents the association of query genes with different GO terms. A lower value of *p* implies more biological significance of the network module. Similarly, *q* value is a statistical measure, which gives the minimum false discovery rate (FDR). In Tables 1 and 2, it is shown that the network modules obtained from Dataset 1 and Dataset 2 have less *p* and *q* values, respectively, which implies the biological significance of our results. Table 1 shows that the modules are biologically enriched with GO terms namely, *cell cycle process, mitotic cell cycle process, chromosomal part, cell cycle, nuclear chromosome part* with 3.801e-24, 5.760e-22, 2.351e-21, 2.188e-20, and 3.413e-18 *p* values, respectively. Table 2 shows that the modules extracted from Dataset 2 are enriched with functions like *gluthathione peroxidate activity, response to wounding, monocarboxylic acid metabolic process, jasmonic acid biosynthetic process, perioxidase activity* with *q* values 2.87e-16, 4.38e-15, 2.10e-12, 5.47e-10, 1.22e-9, respectively. Table 3 gives a comparison between FUMET^4^ and the proposed method in terms of *p* values obtained for Dataset 3. Additionally, Tables 4 and 5 depict that GO terms obtained using the proposed method for Dataset 1 have lower *p* values and *q* values than that of FUMET and Qcut, respectively. This signifies the ability of the method to detect biologically enriched modules with low *p* and *q* values. As shown in Tables 3, 4 and 5, THD-Module Extractor performs better in terms of *p* values and *q* values in comparison to FUMET and Qcut.

### Application of THD-Module Extractor on AD dataset

Application of THD-Module Extractor on AD dataset shows that the method can extract biologically and statistically enriched network modules. As shown in Table 6 and 7, the genes in network modules obtained from AD dataset have lower *p* and *q* values, respectively. For example, in case of Module 1 in Table 6, some of the GO terms, namely, *intracellular organelle part, cytoplasm part organelle, organelle part, intracellular part, membrane bounded organelle* have *p* values 4.920e-47, 8.199e-45, 1.106e-43, 7.388e-40, and 2.785e-39, respectively. Moreover, Module 1 is rich in functions like *cystolic ribosome, translation elongation, nuclear transcribed mRNA catabolic process nonsense mediated decay* with lower *q* values 8.21e-16, 8.80e-16, 1.11e-15, respectively. Therefore, it can be inferred that the network modules extracted from THD-Module Extractor are biologically enriched with GO terms, which have low *p* values and low *q* values with high biological significance.

### Analysis of modules to find genes related to AD

Each network module are extracted from a CEN to find those genes that have less expression similarity with the core gene, yet high semantic similarity with all genes. A gene or a node in CEN *v*_*i*_ ∈ *V* is a core gene iff it is the starting node of a module *m*_*j*_ and satisfies the following conditions.

1. *v*_*i*_ ∉ any other module *m*_*k*_ where *j* ≠ *k*.

2. 
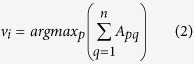


where *A* is the Adjacency matrix, p and q denote the rows and columns in A, and *n* is the total number of genes.

3. nNeighbor (*v*_*i*_) ≥ *ρ*, where nNeighbor (*v*_*i*_) is the total number of *k* genes directly connected to a gene *v*_*i*_, i.e. SSSim (*v*_*i*_, *v*_*k*_) ≥ *δ*.

On the other hand, a gene *v*_*b*_ within a module is a border gene iff ∀*v*_*j*_ ∈ neighbor (*v*_*b*_), SSSim(*v*_*b*_, *v*_*j*_) ≥ *δ* and nNeighbor (*v*_*b*_) < *ρ*. From each biologically enriched modules extracted from the CEN for AD dataset, the border genes are found to have low expression similarity with respective core genes and high semantic similarity among each other. These border genes are then analyzed to find the differentially expressed genes across normal as well as disease samples. The 2000 differentially expressed genes with highest variance across normal and disease samples are considered. These differentially expressed genes are then analyzed using *queryMany()* of *mygene package* in R library to find the entrez IDs of the corresponding genes. The semantic correlations among these entrez IDs are found using *mgeneSim* of *GOSemSim package* in R using Lin’s semantic correlation measure^17^ and Biological Process (BP) structure. In GO-BP structure, genes with annotations are involved with multiple functions^12^. Figure 2 shows an evidence of genes connected in p53 pathway. From the semantic correlation matrix, the genes for which semantic correlations are greater or equal to *β* are extracted, where *β* is the mean semantic correlation score. From this analysis, 912 genes are found to be interesting, with semantic correlation ≥ *β*.

These 912 interesting genes are analyzed using PANTHER and validated using GeneCards. Table 8 provides description of some of the interesting border genes, namely, APBB2, CASP2, CSNK1D, CDK5, HSD17B10, MAPT, PSEN2, and RCAN1. Some of the pathways associated with these border genes are depicted in Table 9 and their description is given in Table 10. A few of these pathways, namely, *Wnt signaling pathway, Alzheimer disease-amyloid secretase pathway, Apoptosis signaling pathway, and Glycolysis* are found to be related to AD, which are further validated using *GeneCards* and existing literature^18^,^19^.

Further, these 912 interesting genes are analyzed to find GO terms related to AD. The GO terms that are found to be related to AD are: *membrane-bounded organelle, negative regulation of apoptotic process, and antigen processing and presentation of peptide antigen via MHC class*, which are validated using *GeneCards*.

Moreover, a Web-based tool called *TopoGSA*^20^ is used to study the topological structures. *TopoGSA* compares the interesting genes against KEGG database and finds KEGG enrichment scores for the given genes. Table 11 depicts that the interesting border genes extracted from the modules, obtained using THD-Module Extractor, are enriched with KEGG pathways. The KEGG pathways *Wnt signaling, Apoptosis, p53 signaling pathway, Notch signaling pathway, Alzheimer’s disease signaling*, associated with the interesting border genes and validated using *GeneCards* and existing literature^18^,^19^, are found to be related to AD. A brief description of the pathways related to AD and the interesting border genes are given in Table 9.

## Discussion

In the past two decades, a good number of methods, such as, FCM^21^, SYNCLUS^22^, FLAME^2^, FUZZY-EWKM^3^,^23^, FUMET^4^, and Qcut^5^ have been introduced for construction of CEN. These methods are based on various correlation measures. Some other CEN methods^24^,^25^,^26^ consider gene CEN, formed by merging networks of heterogenous experiments, specific to plants. Leal *et al*.^24^ introduced a multivariate approach based on Principal Component Analysis (PCA) to study the plant immune responses, by considering gene expression similarity of various plants, namely rice, Arabidopsis thaliana, soybean, cassava and tomato. However, the method proposed herein analyzes genes based on both expression and semantic similarity. The experiments conducted show that the border genes with low expression similarity and high semantic similarity are differentially expressed between normal and disease set of samples with high variance.

The key consideration is that genes or proteins function in co-ordination and not in isolation. The genes or gene products interact in biomolecular network or pathways, which are ultimately associated with the functions of living organisms and other various traits. Therefore, analysis of genes involved in molecular pathway is important for identifying disease-related genes or biomarkers, responsible for dysregulations of functions in disease patients.

AD is a common cause of dementia. In AD patients, it is found that there are some differences in brain tissues in the form of misfolded proteins, known as senile plagues and neurofibrillary tangles. The senile plagues or amyloid plagues are some clumps of *β* amyloid proteins, which block communication between neurons, and implicate neuronal death. The formation of *β* amyloid clumps at early onset of AD is initiated by mutations in proteins, known as Amyloid Beta (A4) Precursor Protein (APP)^27^, Presenilin 1 (PSEN1), and Presenilin 2 (PSEN2) (nlm.nih.gov). On the other hand, neurofibrillary tangles are formed due to mutations in a protein called Tau, which is responsible for ensuring clear passage for food molecules inside microtubule. Mutation in the protein Tau causes disintegration of microtubule and formation of tangles, which obstructs food passage to the cell neurons, resulting in death of neurons^28^. Involvement of genes or proteins in molecular pathways related to AD, such as, *Wnt signaling, p53 signaling, Alzheimer disease-amyloid secretase, Apoptosis signaling, and Glycolysis*, show a close relation with pathogenesis of AD.

In the proposed method, the interesting border genes are extracted from network modules using PANTHER and validated using GeneCards. The border genes are semantically similar as they share some common functionalities in the GO-BP structure, and therefore are found to be involved in molecular pathways. The pathways associated with the border genes related to AD are *Wnt signaling, p53 signaling, Alzheimer disease-amyloid secretase, Apoptosis signaling, and Glycolysis*. Some of the interesting border genes that are found to be related to AD and validated against the existing literature^1^,^29^ are AATF, APBA2, APBB1, APOD, BACE2, CASP2, CAST, CDK5, CSNK1D, GAP43, HSD17B10, KCNIP3, KLK6, MAPT, NQO1, NRGN, OGT, PADI2, PSEN2, and RCAN1. Microtubule-Associated Protein Tau (MAPT) is linked with frontal lobe dementia^29^. PSEN2 is associated with deposition of longer form of amyloid-beta in AD brains, and Caspase 2 (CASP2) is involved in activation of cascade of caspases responsible for apoptosis, which increases cell death^1^. Gene CDK5 is associated with p53 signaling pathway. The p53 signaling pathway is a suppressor of cell growth, i.e., death of neurons in AD patients. Genes, such as, CSNK1D and PSEN2 related to Wnt signaling pathways are found to play an important role in neurodegenerative diseases^30^.

## Method and datasets

**Figure 1:**
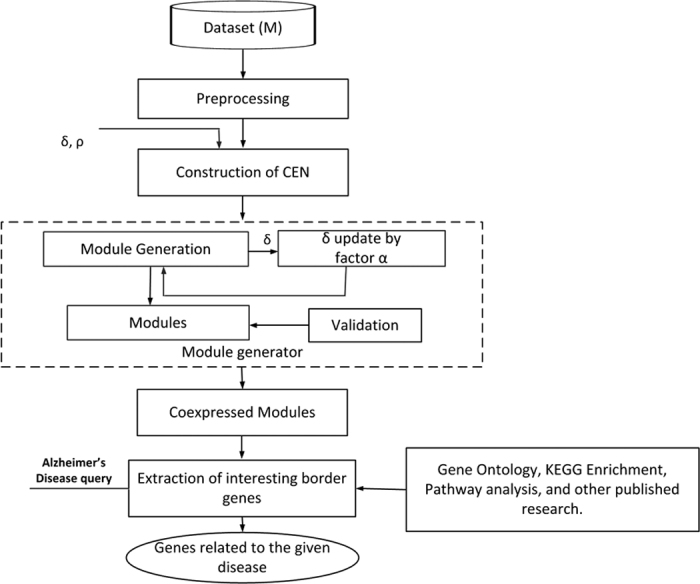
A schematic diagram of THD-Module Extractor framework. The proposed method accepts a microarray gene expression dataset, *M*. After preprocessing, it accepts two thresholds, namely, expression similarity threshold (*δ*) and minimum neighborhood threshold (*ρ*) to construct CEN and to extract modules with high expression similarity using similarity measure, SSSim. THD-Module Extractor updates *δ* by a factor *α* in each iteration of the module extraction process. The method identifies disease related genes by analyzing the border gene set extracted from the highly co-expressed modules and validates them using GO, KEGG Enrichment, pathway analysis, and existing literature.

**Figure 2:**
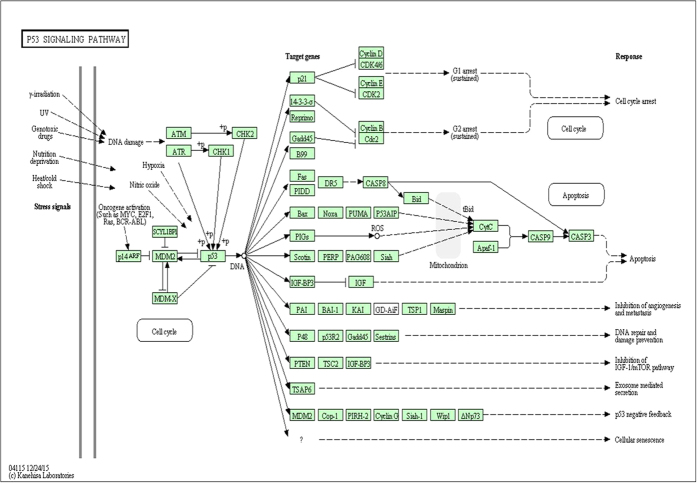
An evidence of p53 signaling pathway obtained using the DAVID tool from KEGG database 32,33**, under CC BY.** Some of the genes, such as PIGs, CDK4/6, CASP9, IGF-BP3, GADD45 invovled in the pathway are found to be related to Alzheimer’s disease.

The proposed THD-Module Extractor method is implemented using Matlab R2015a on a machine with an Intel(R) core I3-2120 CPU@3.30Ghz processor, a 2.00 GB RAM, and a 64-bit Windows 7 operating system. The algorithm is evaluated on four datasets. The description of the datasets is given in Table 12. Figure 1 depicts the work flow of the method. The various steps of execution are detailed below.

### Preprocessing

Preprocessing of datasets is important before implementation of any scientific algorithm. Many datasets contain noisy values and NULL values. The following preprocessing tasks are performed on the considered datasets using Matlab, before storing them in *tab delimited* form.

- The expression values with unlabeled or unformatted^28^ genes are removed.
- Genes having only undefined (NaN) expression value(s) are removed.
- Redundant or duplicate genes are removed.

The AD dataset GSE4226 consists of blood mononuclear cells collected from AD patients and age-gender matched normal individuals with 9600 probes and 28 samples. This work considers perturbation of gene expression values during progression of the disease, from normal to disease stage. Therefore, expression values of probes for AD patients and normal individuals are considered and rest are discarded.

After preprocessing of the datasets, the SSSim score of each gene pair is computed. However, computation of SSSim score for each gene pair takes lots of execution time. The datasets considered are large, often containing several thousands of genes, each with many expression values for multiple varying conditions. In our case, the preprocessed AD dataset contains expression values of more than 9000 genes under 28 different conditions, even after removing the unnecessary entries. This requires computation of more than 81 million getSSSim values. Traditional implementation of the measure on a standard CPU requires a huge amount of processing time. Therefore, this work leverages the parallel processing capabilities of GPU to speed up the computation of the large getSSSim correlation matrix.

### NVIDIA CUDA implementation for SSSim computation

**Figure 3:**
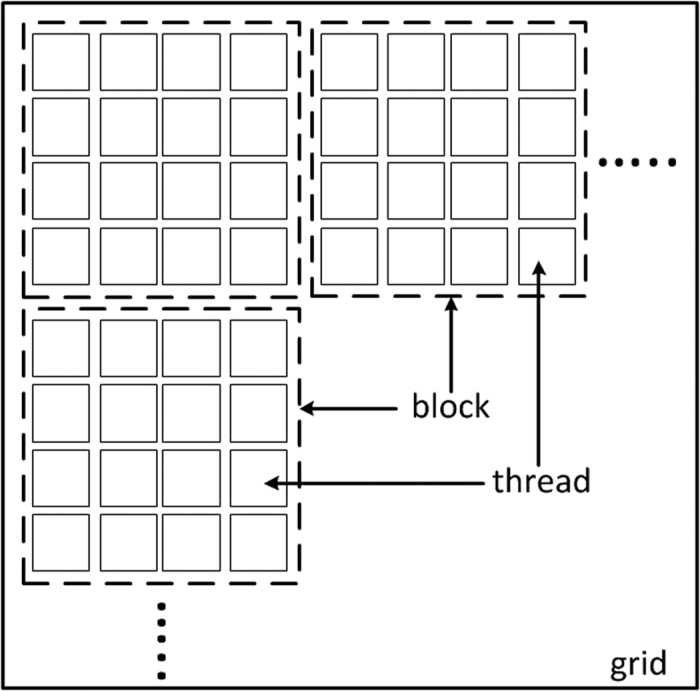
GPU grid configuration for getSSSim. Computation of the getSSSim matrix is expressed as a large grid in the GPU kernel. The grid contains GPU threads equal to the number of getSSSim values to be calculated. The organization of the grid is hierarchical. The grid is a 2D array of blocks and each block is a 3D array of threads. For simplicity, the blocks are shown as 2D arrays of threads.

The NVIDIA CUDA 7.5 API and its C/C++ based compiler (NVCC) are used to implement the getSSSim correlation matrix computation on GPU. An NVIDIA GeForce 980 GPU with compute capability 5.2^31^ is used in this implementation. The computation of the getSSSim correlation matrix is distributed into a logical 2-D grid of thread blocks, where each thread processes one getSSSim value. The dimension of each thread block is 4 × 4 × 28, which specifies the number of threads per block. The dimension of the grid is 2271 × 2271, which specifies the number of blocks in a grid. Figure 3 shows the distribution of threads into the grid. The GPU used in our implementation contains 2048 cores and can process equal number of threads simultaneously. In case of the AD dataset, the speedup obtained using the GPU for the correlation matrix computation is more than 1200 times, in comparison to a Matlab based implementation using a standard Intel-i7 CPU.

Once the score matrix has been computed, the CEN is constructed from the adjacency matrix, *A*. From the CEN, co-expressed modules are extracted for different values of *δ* and a static *ρ*. Each module contains a core gene that has maximum number of neighbor genes, satisfying the expression similarity threshold *δ* within the module. These co-expressed modules are then validated in terms of external validation metrics. A Web-based tool FuncAssociate 2.0 is used to find *p* values of each GO term associated with the genes of the co-expressed modules. Another Web-based tool called GeneMania^1^ is used to find the *q* values of each GO term. From each co-expressed module extracted from AD dataset, the border genes that satisfy the expression similarity threshold *δ* with their neighbors, but not the minimum neighborhood threshold *ρ*, are found.

### THD-Module Extractor in finding interesting genes for AD

In addition to biologically relevant network module extraction, the proposed THD-Module Extractor allows identification of disease related genes by analyzing the border genes, extracted from the highly co-expressed modules. These border genes are analyzed using R library. Among the border genes, the *k* differentially expressed genes with highest variance between normal and disease samples are considered. Further, the entrez ID of each differentially expressed gene is found using *mygene package* of R. Thereafter, the semantic similarity of each of these border genes among each other is computed using *GOSemSim package* of R. On the basis of a static threshold, the border genes with high semantic similarity are filtered out. These interesting border genes are found to have low expression similarity with the core genes, yet high semantic similarity among each other. These interesting border genes are then validated using pathway analysis, KEGG enrichment analysis, GO terms enrichment analysis, and existing literature.

## Additional Information

**How to cite this article**: Kakati, T. *et al*. THD-Module Extractor: An Application for CEN Module Extraction and Interesting Gene Identification for Alzheimer's Disease. *Sci. Rep.* **6**, 38046; doi: 10.1038/srep38046 (2016).

**Publisher's note:** Springer Nature remains neutral with regard to jurisdictional claims in published maps and institutional affiliations.

## Figures and Tables

**Table 1:** *p* values of network modules for Dataset 1 (Subset of yeast cell cycle).

Module No.	Number of genes	Gene Ontology ID	GeneOntology Attribute	*p* value
Module 1	284 genes	GO:0022402	cell cycle process	3.801e-24
		GO:1903047	mitotic cell cycle process	5.760e-22
		GO:0044427	chromosomal part	2.351e-21
		GO:0007049	cell cycle	2.188e-20
		GO:0044454	nuclear chromosome part	3.413e-18
Module 2	97 genes	GO:0022402	cell cycle process	2.227e-14
		GO:1903047	mitotic cell cycle process	6.703e-14
		GO:0007049	cell cycle	2.438e-13
		GO:0000280	nuclear division	3.322e-9
Module 3	89 genes	GO:0022402	cell cycle process	3.875e-14
		GO:0007049	cell cycle	1.537e-13
		GO:103047	mitotic cell cycle process	3.998e-13
		GO:0000280	nuclear division	8.879e-10
		GO:0048285	organelle fission	2.103e-9

**Table 2:** *q* values of network modules for Dataset 2 (Arabidopsis thaliana).

Module No.	Number of genes	Functions	*q* value (FDR)
Module 1	70 genes	gluthathione peroxidate activity	2.87e-16
		response to wounding	4.38e-15
		monocarboxylic acid metabloic process	2.1e-12
		jasmonic acid biosynthetic process	5.47e-10
		perioxidase activity	1.22e-9
Module 2	16 genes	apoplast	2.40e-7
		transferase activity transfering alkyl or aryl groups	1.56e-3
		response to cold	5.57e-3
		cellular response to water stimulus	6.64e-3
		copper ion binding	6.64e-3
Module 3	27 genes	aromatic amino acid family biosynthetic process	2.35e-9
		aromatic amino acid family metabolic process	5.38e-9
		cellular amino acid biosynthetic process	8.36e-6
		monocarboxylic acid metabolic process	1.09e-5
		lyase activity intramolecular transferase activity	2.91e-5
		indolalkylamine biosynthetic process	2.94e-5

**Table 3:** Comparison of THD-Module Extractor with FUMET in terms of *p* values for Dataset 3 (Yeast sporulation)^4^.

GO_ID	GO attribute	THD-Module Extractor	FUMET
GO:0048646	Anatomical structure formation	7.771e-36	3.38e-31
GO:0043934	Sporulation	2.478e-34	1.060e-25
GO:0030154	Cell Differentiation	1.757e-33	1.06e-25
GO:0030435	Sporulation resulting in formation of a cellular spore	1.757e-33	1.43e-25
GO:0032505	Developmental process	4.132e-28	2.06e-22
GO:0048869	Cellular developmental process	4.002e-27	3.13e-23
GO:0005628	Prospore membrane	8.597e-21	5.72e-16

**Table 4:** Comparison of THD-Module Extractor with FUMET and Qcut in terms of *p* values for Dataset 1^4^.

GO_ID	GO attribute	THD-Module Extractor	FUMET	Qcut
GO:0022402	Cell Cycle process	3.801e-24	1.482e-13	6.585e-8
GO:0044427	Chromosomal part	2.351e-21	7.860e-10	2.274e-9
GO:0007049	Cell Cycle	2.188e-20	5.388e-14	1.165e-7
GO:0044454	Nuclear chromosome part	3.413e-18	7.860e-10	2.274e-9
GO:0006281	DNA repair	1.949e-16	3.422e-9	2.970e-8
GO:0006974	Response to DNA damage stimulus	9.465e-16	1.461e-9	2.983e-7
GO:0051301	Cell division	2.508e-15	2.619e-7	1.547e-6
GO:0051716	Cellular response to stimulus	1.662e-10	6.375e-8	1.872e-7
GO:0006260	DNA replication	2.153e-13	2.124e-8	1.082e-7
GO:0006270	DNA replication initiation	1.688e-8	2.360e-8	1.081e-7
GO:0048523	Negative regulation of cellular process	5.185e-8	8.852e-9	7.970e-7
GO:0048519	Negative regulation of biological process	4.719e-8	1.308e-8	1.073e-6
GO:0005634	Nucleus	8.278e-8	4.693e-9	3.967e-8

**Table 5:** Comparison of results obtained from THD-Module Extractor with FUMET and Qcut in terms of *q* values for Dataset 1^4^.

GO Annotation	THD-Module Extractor	FUMET	Qcut
DNA repair	1.97e-20	7.87e-14	5.25e-13
Nuclear chromosome part	3.76e-17	1.65e-13	1.02e-12
Nuclear chromosome	5.19e-18	1.05e-14	2.41e-13
Cellular bud	2.65e-7	6.22e-9	4.07e-3
Replication fork	8.43e-19	8.852e-11	1.78e-10
Mitosis	3.86e-8	4.12e-8	1.49e-5

**Table 6:** *p* values and *q* values of the network modules for Dataset 4 (AD).

Module no.	Size of module	GO_ID	GO attribute	*p* value	Function	*q* value
1	3652 genes	GO:0044446	intracellular organelle part	4.920e-47	cystolic ribosome	8.21e-16
		GO:0044444	cytoplasm part organelle	8.199e-45	translational termination	8.21e-16
		GO:0044422	organelle part	1.106e-43	translational elongation	8.8e-16
		GO:0044424	intracellular part	7.388e-40	nuclear transcribedmRNA catabolic process, nonsense-mediated decay	1.11e-15
		GO:0043227	membrane-bounded organelle	2.785e-39	viral gene expression	1.11e-15
		GO:0043229	intracellular organelle	1.767e-34	viral trascription	2.81e-15
		GO:0010467	gene expression	3.941e-26	ribosomal subunit	2.81e-15
		GO:0044403	symbiosis, encompassing mutualism through parasitism	2.279e-20	multi-organism metabolic process	7.27e-15
		GO:0043604	amide biosynthetic process	1.951e-18	SRP-dependent cotranslational protein targeting to membrane	1.33e-14
		GO:0006614	SRP-dependent cotranslation protein targeting to membrane	2.059e-18	cotranslational protein targeting to membrane	1.40e-14

**Table 7:** *p* values and *q* values of the network modules for Dataset 4 (continued).

Module no.	Size of module	GO_ID	GO attribute	*p* value	Function	*q* value
Module 2	442 genes	GO:0003723	RNA Binding	3.226e-10	translational initiation	3.81e-8
		GO:0044444	intracellular membrane-bounded organelle	3.357e-8	translational elongation	8.05e-7
		GO:0043231	negative regulation of organelle organization	4.805e-8	protein localization to endoplasmic reticulum	4.44e-6
		GO:0005829	cytosol	6.028e-8	viral life cycle	7.37e-6
		GO:0032991	macromolecular complex	7.931e-8	nuclear transcribed mRNA catabolic process, nonsense-mediated decay	1.46e-5
		GO:0051129	negative regulation of cellular component organization	2.187e-7	SRP-dependent cotranslational protein targeting to membrane	2.43e-5
Module 3	209 genes	GO:0031982	vesicle	1.950e-7	protein polyubiquitination	2.19e-2
		GO:0031968	memebrane-bounded vesicle	2.300e-7	DNA damage response, signal transduction by p53 class mediator resulting in cell cycle arrest	2.19e-2
		GO:0065010	extracellular memebrane-bounded organelle	3.840e-7	signal transduction involved in mitotic cell cycle checkpoint	2.19e-2
		GO:0070062	extracellular exosome	3.840e-7	signal transduction involved in mitotic DNA damage checkpoint	2.19e-2
		GO:0043230	extracellular organelle	4.521e-7	cell cycle arrest	2.19e-2
		GO:1903561	extracellular vesicle	4.521e-7	signal transduction involved in DNA integrity checkpoint	2.22e-2
		GO:0002684	positive regulation of immune system process	3.55e-5	G1 DNA damage checkpoint	2.72e-2
		GO:0002757	immune response activating signal transduction	9.184e-4	antigen processing and presentation of exogenous peptide antigen	2.72e-2

**Table 8:** Some Interesting Border Genes and their Description.

Gene name	Description	Gene name	Description
APBB2	Mutation in APBB2 (Amyloid Beta (A4) Precursor Protein-Binding, Family B, Member 2) causes Alzheimer’s disease^1^	CASP2	Caspase 2 is involved in activation of cascade of caspases responsible for apoptosis, which increases the cell death^1^
CSNK1D	Casein kinase 1 delta protein is involved in apoptosis, which regulates degeneration or cell death during progresssion of AD^34^	CDK5	Cyclin-dependent kinase 5 enhances A*β* production or A*β* amyloidgenesis, which is seen in AD pateints during progression stages^35^
HSD17B10	Mutation in Hydroxysteroid (17-Beta) Dehydrogenase 10 is responsible for mental retardness in male^36^	MAPT	Microtubule-Associated Protein Tau (MAPT) is associated with Pick disease type taupathy, which is linked with frontal lobe dementia^29^
PSEN2	Mutations in Presenilin 2 (PSEN2) is a key initiator for formation of amyloid plagues (nlm.nih.gov)	RCAN1	Regulator Of Calcineurin 1 activates CASP3 and induces apoptosis^37^

**Table 9:** Pathways associated with interesting border genes obtained from Dataset 4.

Pathway Accession Number	Pathways
P00004	Alzheimer disease-presenilin pathway
P00059	p53 pathway
P05912	Dopamine receptor mediated signaling pathway
P00045	Notch signaling pathway
P06587	Nicotine pharmacodynamics pathway
P00003	Alzheimer disease-amyloid secretase pathway
P00049	Parkinson disease
P00015	Circadian clock system
P06959	CCKR signaling map
P00007	Axon guidance mediated by semaphorins
P00057	Wnt signaling pathway

**Table 10:** Analysis of the pathways related to AD^1^.

Pathway name	Description
Apoptosis Pathway	This pathway is responsible for death of cells. Since Alzheimer’s disease is associated with damage of neurons, so Apoptosis has a major role in Alzheimer’s disease^38^. Genes like MAPT, CASP2 catalyzes Apoptosis.
Notch Pathway	This pathway is responsible for maturation, cell division, and functioning of immune system. Genes like PSEN1, MYC, PSEN2 are associated with Notch pathway. In patients with Alzheimer’s disease, there is mutation in PSEN1 or PSEN2, which leads to deposition of longer form of amyloid beta in AD brains.
Wnt Pathway	Wnt plays an important role in development of neurons in adult brain and this implicate in neurodegenerative disease^30^. Genes like CSNK1D, DKK1, PIN1, PSEN1, PSEN2 are related to Wnt signaling pathways.
p53 signaling pathway	The p53 tumor suppressor gene is a key gene for apoptosis and activates cell growth. Since death of neuron is associated with Alzheimer’s disease, so p53 signaling pathway is an important regulator of Alzheimer’s disease. Genes like CASP3, CDK5, CREB1 are related to p53 signaling pathway.

**Table 11:** Quality scores of KEGG pathways associated with interesting gene sets of Dataset 4.

Identifier	Median degree	Median CC	Median SPL	Median BW	Median EVC	No. of genes	Score
hsa05220:Chronic myeloid leukemia	36	0.08	3.3	49218	0.1	76 (0)	0.97
hsa05212:Pancreatic cancer	30	0.07	3.35	45788	0.07	75 (0)	0.93
hsa05223:Non-small cell lung cancer	29	0.07	3.35	38253	0.08	55 (0)	0.93
hsa04320:Dorso-ventral axis formation	30	0.06	3.32	32814	0.1	22 (0)	0.92
hsa05221:Acute myeloid leukemia	27	0.09	3.39	32521	0.07	57 (0)	0.92
hsa05215:Prostate cancer	28	0.06	3.37	35660	0.07	84 (0)	0.9
hsa05040:Huntington’s disease	31	0.03	3.35	59658	0.05	28 (0)	0.85
hsa04210:Apoptosis	21	0.09	3.51	21503	0.04	81 (0)	0.84
hsa04115:p53 signaling pathway	21	0.07	3.57	18855	0.02	59 (0)	0.78
hsa04330:Notch signaling pathway	15	0.08	3.59	11534	0.03	40 (0)	0.74
hsa04370:VEGF signaling pathway	16	0.04	3.5	15764	0.04	63 (0)	0.73
hsa04510:Focal adhesion	15	0.06	3.55	14237	0.03	181 (1)	0.72
hsa04910:Insulin signaling pathway	14	0.07	3.58	14485	0.03	123 (2)	0.72
hsa05010:Alzheimer’s disease	16	0.05	3.63	24871	0.02	28 (1)	0.71
hsa05020:Parkinson’s disease	9	0.03	3.74	13520	0.01	20 (2)	0.55
hsa04310:Wnt signaling pathway	10	0.04	3.76	9607	0.01	123 (1)	0.54

**Table 12:** Datasets and their description.

Serial no.	Dataset	Size of dataset	Source
1	Subset of yeast cell cycle	384 × 17	http://faculty.washington.edu/kayee/cluster
2	Arabidopsis thaliana	138 × 8	http://homes.esat.kuleuven.be/sistawww/bioi/thijs/Work/Clustering.html
3	Yeast sporulation	474 × 17	http://cmgm.stanford.edu/pbrown/sporulation
4	Homo sapiens (AD)	9084 × 28	http://agnigarh.tezu.ernet.in/~dkb/resources.html
